# Application and Analysis of Discrete Fiber Probes in Determining Detonation Velocity of Microcharges

**DOI:** 10.3390/mi12121524

**Published:** 2021-12-08

**Authors:** Guodong Zhang, Yulong Zhao, Jing Sun

**Affiliations:** State Key Laboratory for Manufacturing Systems Engineering, Xi’an Jiaotong University, Xi’an 710054, China; zgd20190624@xjtu.edu.cn (G.Z.); sun543927@stu.xjtu.edu.cn (J.S.)

**Keywords:** detonation velocity, microcharges, fiber probe

## Abstract

This paper describes a method based on discrete fiber probes for measuring detonation velocity produced by microcharges. This method is simple to implement, scalable for multi-channel and requires minimal perturbation to the detonation wave. A simple experimental apparatus was established by using the oscilloscope, photodetectors, optical fibers, alignment device and initiation system. Four groups of experiments were carried out for analyzing the influence of probe spacing on detonation velocity. The experiment results suggest that a relatively appropriate distance between two adjacent fiber probes is 4 mm. In addition, the comparative experiments between ionization probes and fiber probes were performed, which shows that the standard deviation of detonation velocity obtained by fiber probes is smaller under the same measurement conditions. This research may be useful for the development of determining detonation velocity precisely of microcharges.

## 1. Introduction

With the development of microelectromechanical systems (MEMS) initiating explosive devices and MEMS fuses, charge designs are approaching minimum dimensions at which many explosives can function. MEMS technology such as direct ink writing has been gradually applied for realizing the manufacture of microcharges with millimeter or sub-millimeter level. However, the charge dimension is related to the diameter effect. This effect shows that every explosive has a critical diameter below which detonation wave will not propagate and a limiting diameter upon which detonation velocity will not increase. The diameter effect curve is nearly relational with many factors, including explosive type, charge density and particle size of explosive. The curve has practical importance, for example, it can be used as an engineering tool for gauging the size of the system in which an explosive will behave ideally [1]. In order to achieve accurately the diameter effect curve, it is imperative to study the method for determining the detonation velocity of microcharges.

To date, much attention has been devoted to detonation velocity measurement technology. Benterou et al. [2] presented a detector of detonation velocity using a 125 μm diameter optical fiber with an integral chirped fiber Bragg grating as an intrinsic sensor and obtained detonation velocity of a 25.4 mm diameter PBX-9502. Vuppuluri et al. [3] reported microwave interferometry that can determine detonation velocity with the dielectric constant of explosives and the instantaneous phase difference of reflected microwave signals from the detonation front. They compared detonation velocity between a novel co-crystal of MDNT and CL-20 of 6.35 mm diameter and a physical mixture of MDNT and CL-20 in the same molar ratio and charge size. Hare et al. [4] introduced an embedded fiber optic probe to directly get detonation velocity continuously in time by measuring the Doppler shift of laser light reflected from the jump caused by the refractive index discontinuity. Their observations were carried out on PBX-9502 and LX-17 of 25.4 mm diameter at varying initial charge density. Sheffield et al. [5] designed an in situ magnetic gauging based on Faraday’s law which needs to be embedded in the sample at an angle and investigated the detonation velocity of PBX-9501 of 51 mm diameter. Li et al. [6] developed a pressure-conducted type of novel velocity probe whose length is continuously shortened as the detonation wave travels through the explosive charge. They implemented a detonation velocity measurement for porous prilled ANFO of different densities with a diameter of 40 mm.

However, the aforementioned detonation velocity measurement methods are mainly used for large-size explosives. In contrast, little work has been focused on the detonation velocity measurement of microcharges with charge diameter of sub-millimeter level. Jung et al. [7] employed streak camera photography to document the firing of lead azide and silver azide contained in micro-capillaries at sub-millimeter. The resulting image on the film has a time-resolved horizontal axis and a position-resolved vertical axis. A straight line on a streak image corresponds to a reaction proceeding and its slope denotes the steady detonation velocity. However, streak camera photography is limited to an explosive with a transparent constraint shell or naked explosive charge. In addition, it is difficult to make the measurement accuracy greater than 1% due to the complexity of calibrating the spatial dimension on the film in microscale and the position uncertainty of the straight line displayed in the streak image [8]. Li et al. [9] designed a membrane sensor with six groups of discrete ionization probes for studying the diameter effect of superfine insensitive HMX charged in small-sized channels. The sensor was placed on the surface of the explosive to reduce the disturbance to detonation waves. Nevertheless, discrete ionization probes are susceptible to electromagnetic interference and hard to respond to weak ionization signals.

Considering that the detonation propagation process of the microcharge is easily disturbed, the embedded approaches including optical fiber based on chirped Bragg grating, continuous velocity probe, and particle velocity gauge, are not suitable for measurement on a microscale. As to microwave interferometry, it is hard to miniaturize the wave-guide to match the microcharge size and microwave energy is generally attenuated by solid explosives, requiring relatively powerful and expensive microwave interferometer to generate extremely high-frequency signals [10].

The objective of the present paper is to explore a simple and practical method for determining the detonation velocity of microcharges based on discrete optical fiber probes. The optical fiber is extremely resistant to electromagnetic interference, high temperature, and pressure environment. Moreover, it is easy to set up because of its good flexibility and low cost. In this contribution, the detonation velocity measurement method of microcharges is established, the influence of probe spacing on detonation velocity is analyzed, and the comparison experiments of detonation velocity measurement with fiber probes and ionization probes were conducted.

## 2. Method

### 2.1. Experimental Principle

In the process of explosive detonation, detonation products near the detonation wavefront can emit strong light radiation because they are in a state of high temperature and high pressure. The radiation contains infrared, visible, and ultraviolet light, and covers the wavelength from 10 nm to 1 mm [11]. In other words, optical signals are generated continuously with the propagation of the detonation wave. Therefore, the detonation velocity of the explosive can be obtained by detecting the relative arrival time of the optical signal at certain positions.

**Figure 1:**
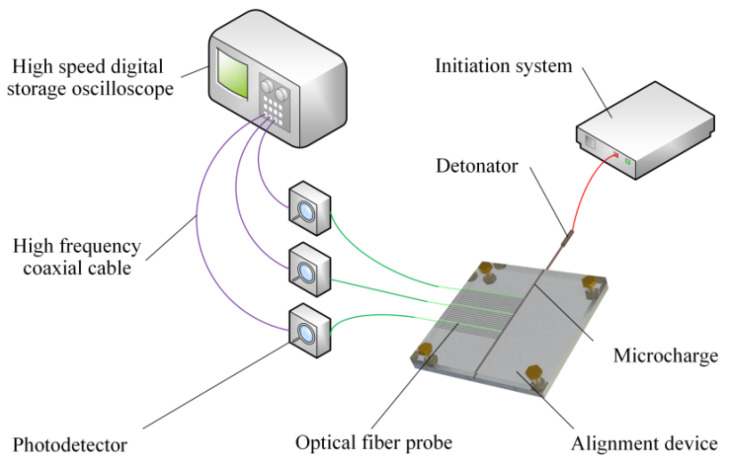
Schematic diagram of the experimental apparatus.

**Figure 2:**
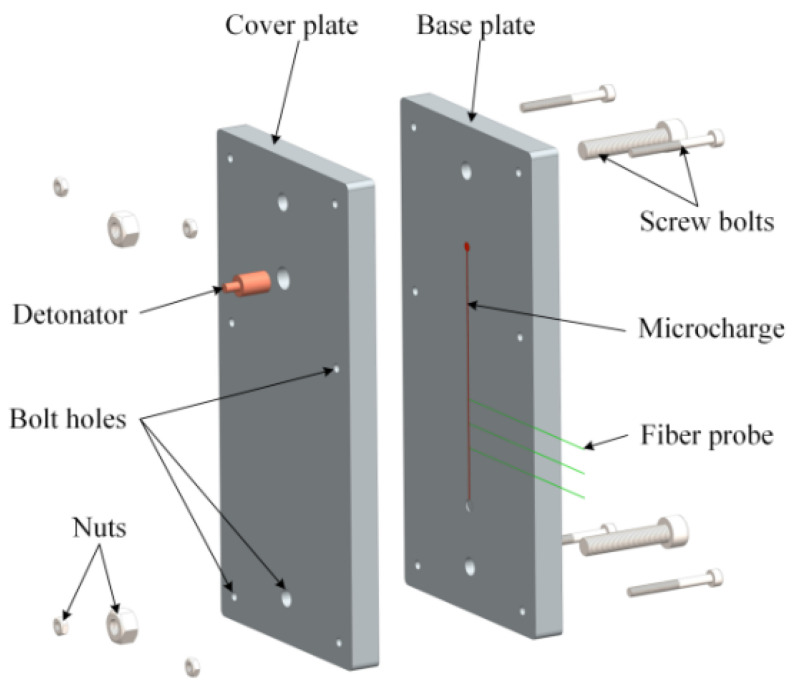
Schematic diagram of the alignment device.

According to the above principle, an experimental apparatus employed for detonation velocity measurement of microcharges is established and its schematic diagram is shown in Figure 1. The apparatus fundamentally consists of a high-speed digital storage oscilloscope, high-frequency coaxial cables, photodetectors, optical fiber probes, alignment device, microcharges, a detonator, and an initiation system. As shown in Figure 2, the alignment device is composed of a base plate and a cover plate, and they are made of aluminum for meeting the constraint condition of microcharges and fixed with screws. The detonator hole was set to align the detonator with the initiation end of the explosive. Bare optical fibers as the probes were placed in the grooves of the base plate at a certain spacing *L*. The direction of the grooves is perpendicular to the propagation direction of the detonation wave and the depth of the grooves is designed to ensure alignment between the center of the probe and the longitudinal section of the microcharge. The bare end of every optical fiber was in close contact with the charge surface. The center of the detonator hole is 30 mm away from the first optical fiber probe, which can avoid the interference of the detonator initiation to the probe and ensure that the measured section is stable detonation.

The microcharge is detonated by the output pressure of the detonator ignited by the initiation system. The optical signals produced by detonation and transmitted through optical fibers are fed successively to photodetectors and then converted to electrical signals with time sequence. The electrical signals are displayed on high-speed digital storage oscilloscope through high-frequency coaxial cable. The time interval Δ*T* between the electrical signals is read from the oscilloscope. Consequently, average detonation velocity can be written as

(1) $$\bar{D}=\frac{L}{\Delta T}.$$

Under the condition of given probe spacing, when the experimental system is used to measure different detonation velocities, the time interval of signals collected by the oscilloscope is also different. Therefore, the system resolution mainly depends on the sample interval of the oscilloscope. Note that it is more reliable to use the electrical signal of the first probe as the trigger signal of the oscilloscope rather than the initiation current. The initiation time of the detonator is millisecond level and the time interval Δ*T* is sub-microsecond level. The time base of the oscilloscope is small under the condition of a high sampling rate. When the initiation current is used as the trigger signal, the electrical signals may not be acquired within the scope of the oscilloscope screen.

### 2.2. Choice of Optical Fiber

**Figure 3:**
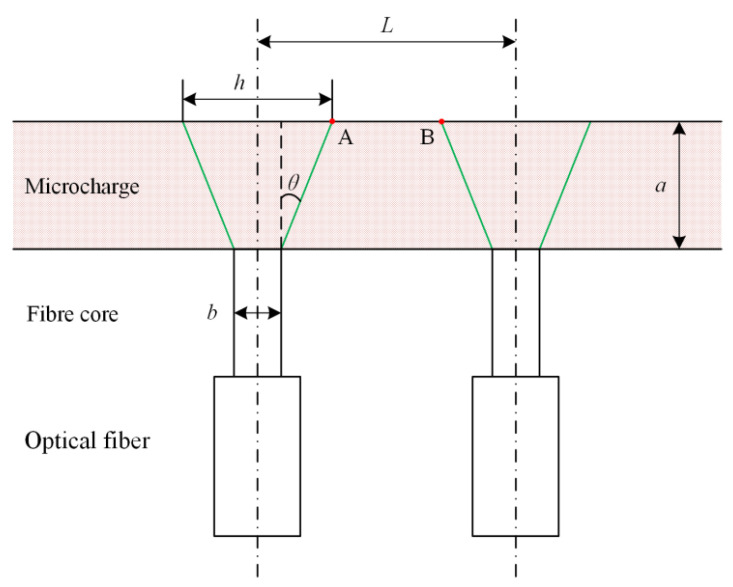
Diagrammatic sketch of the probes’ arrangement.

The step-index multimode optical fiber is employed in this paper. The main structure parameters of optical fiber are the core diameter and numerical aperture (NA). The NA represents the angle range of incident light received by the fiber and its value of multimode fiber ranges from 0.18 to 0.23, which is suggested by CCITT. For collecting more optical signals, the NA of the fiber used in this experiment is set to 0.22. In the longitudinal section of the microcharge and optical fiber, due to the existence of NA, the maximum light-receiving range projected to the charge boundary is defined as *h*, as shown in Figure 3. When point A and point B coincide, the fiber spacing is represented as *L*’ which is numerically equal to *h*. During the experiment, the fiber spacing *L* must be greater than *L*′ for avoiding mutual interference,

(2) $$L>L^{\prime }=h=\frac{2a\cdot \mathrm{NA}}{\sqrt{1-{\mathrm{NA}}^{2}}}+b,$$

where *a* is charge width, *b* denotes core diameter, and NA = sin *θ* = 0.22.

In addition, the larger the core diameter, the greater the optical power coupled into the fiber, which will increase the output voltage of the photodetector. However, according to Equation (2), a large core diameter is not conducive to the dense arrangement of probes. Therefore, through comprehensive consideration of the above factors, the core diameter of the fiber used in this experiment is set to 105 μm.

The chromatic dispersion is attributed to the variation of the propagation time of optical signals in fiber with frequencies and mode types and it results in pulse broadening which will affect the rise time of oscilloscope signals. In order to reduce the influence of fiber dispersion on the optical signals, the optical fiber should be as short as possible, preferably no more than 20 m [12]. Combining with the experimental requirements, the length of every optical fiber is set to 10 m. For the step-index multimode optical fiber, mode dispersion needs to be mainly considered. In these conditions, the delay skew caused by mode dispersion is ~0.53 ns. Moreover, because the time interval between two rising edges of the oscilloscope signal is concerned in this paper, the influence of dispersion on the measurement results can be ignored.

Based on the above analysis, the step-index multimode optical fibers were commercially obtained, and their structural parameters are given in Table 1. The NA and core diameter are used to calculate the *h* value and the groove of the base plate is designed as 0.3 mm width according to the coating diameter.

### 2.3. Choice of Photodetector

In order to facilitate reading the time signal, the photodetector should possess short rise time, high sensitivity, and high signal-to-noise ratio. In this paper, three kinds of photodetectors are compared for observing their response to the same optical signal produced by microcharge detonation and obtaining an appropriate one for this paper. Their performance parameters are given in Table 2. The photodetector of FPD310-FC-VIS incorporates an RF amplifier, the photodetector of KG-PR-500M-B includes a trans-impedance amplifier, and the photodetector of DET025AFC/M has no internal gain.

**Figure 4:**
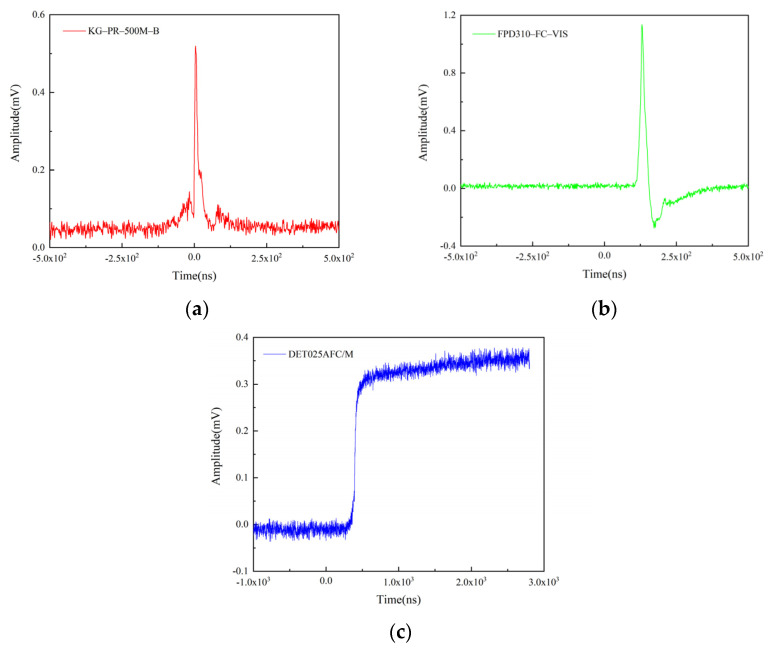
Typical signals of: (**a**) KG-PR-500M-B, (**b**) FPD310-FC-VIS, and (**c**) DET025AFC/M.

Under the same detonation experiment conditions such as a booster explosive of 0.5 mm width × 0.5 mm high, the typical signals corresponding to the three photodetectors are shown in Figure 4. Note that the rising processes of FPD310-FC-VIS and DET025AFC/M have less noise, which is conducive to reading the time signal. However, the photodetector of DET025AFC/M offers a shorter rise time and lower cost. Furthermore, the experimental results show that the incident light power does not exceed the damage threshold of the photodetector. Therefore, the DET025AFC/M photodetector is employed in this paper.

## 3. Experiment

### 3.1. Microcharges

**Figure 5:**
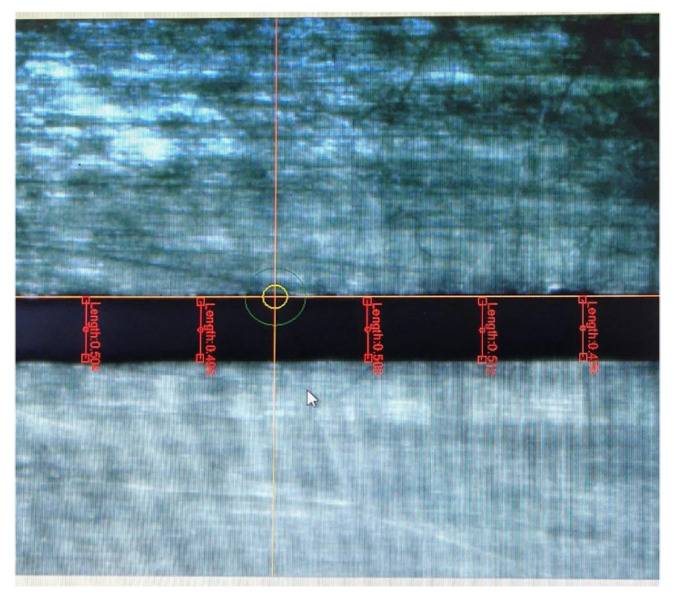
Charge width measurement.

In this part, the selected optical fiber was used for measuring the detonation velocity generated by a booster explosive. The charge groove designed as 0.5 mm width and 0.5 mm high is depicted in Figure 5. The booster explosive was charged into the groove by direct ink writing technology [13]. The charge width is a key parameter and it can affect the detonation velocity in two ways: first, when the charge width decreases, the lateral energy loss of the detonation process increases, resulting in the decrease of detonation velocity, and second, the value of *h* in Equation (2) is subject to the charge width, which may influence the measurement uncertainty of detonation velocity. In the charge length direction, five points were selected for width measurement by utilizing the microscopic measurement function of the inkjet printer and the results are listed in Table 3.

### 3.2. Experimental Procedure

During the experiment, the following procedures must be strictly executed for safety and reliability.

**Figure 6:**
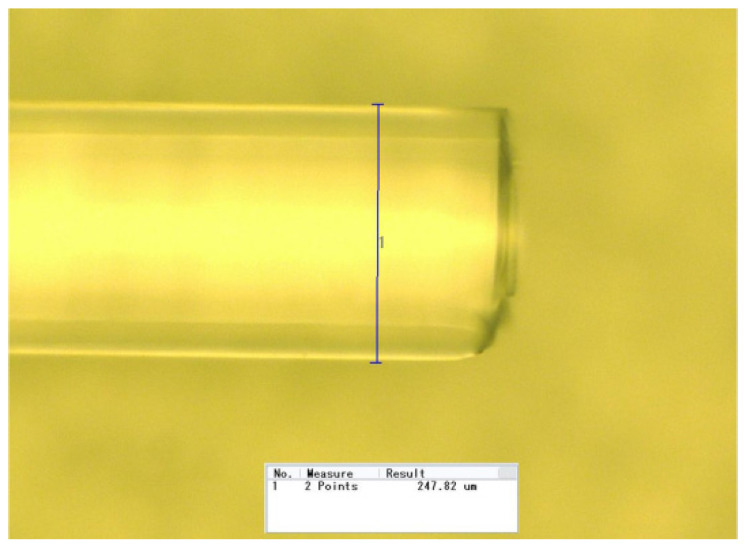
Microscope figure of the optical fiber cut by optical fiber cleaver.

**Figure 7:**
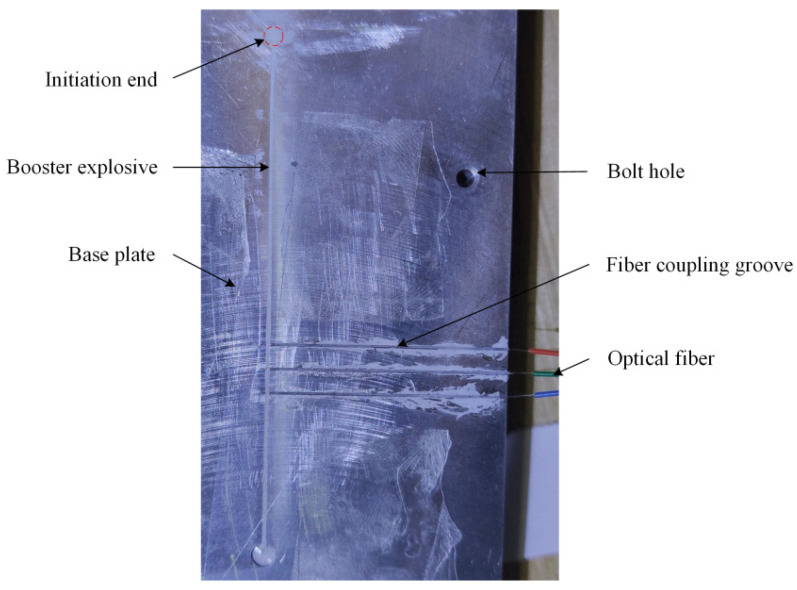
The installation site.

**Figure 8:**
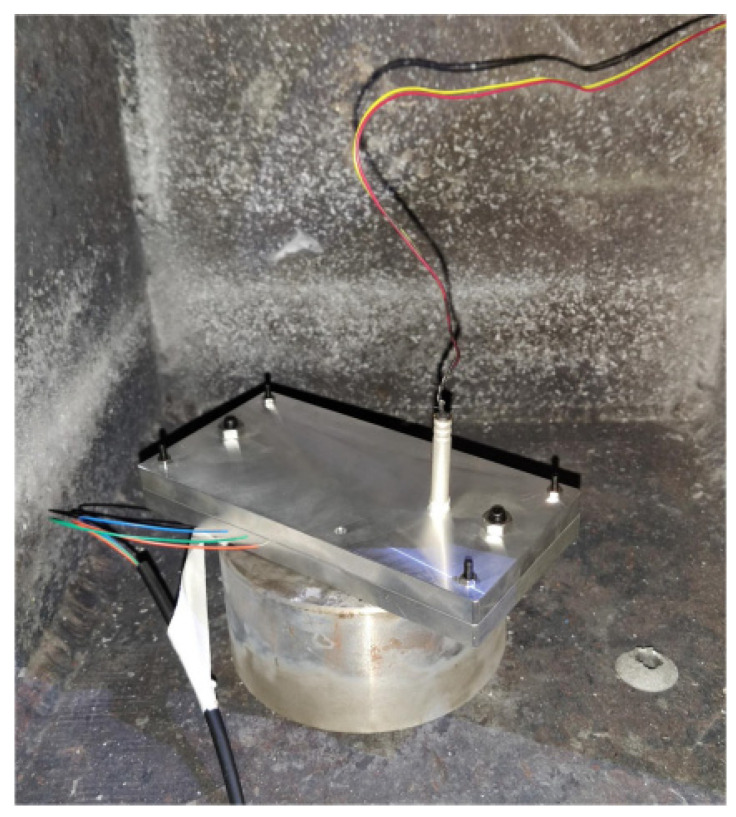
Assembled apparatus for detonation velocity measurement.

(1) As shown in Figure 6, the bare end of the optical fiber was cut through an optical fiber cleaver to avoid reducing the maximum receiving angle of optical signals, and then the optical power meter was used to check whether the whole fiber is in good condition.
(2) The other end of the optical fiber was connected with a photodetector, and then the photodetector was attached to the oscilloscope through high-frequency coaxial cable and powered by a battery.
(3) The oscilloscope parameters were set, and the signal circuit was checked by utilizing the optical power meter.
(4) The bare ends were placed in fiber coupling grooves of the base plate during which fiber end faces must be fixed against the charge, as Figure 7 shows.
(5) The base plate and cover plate were bolted with an electric screwdriver at a fixed torque.
(6) As shown in Figure 8, the assembled apparatus was placed in the small explosion vessel, and then the detonator was inserted into the detonator hole of the cover plate and connected with the initiation system.
(7) Checking the circuits, if everything is normal, then the detonator was ignited and time values were read from the oscilloscope (Tektronix DPO 4045 with a bandwidth of 500 MHz and sampling rate of 2.5 GS/s).

### 3.3. Detonation Velocity Calculation

**Figure 9:**
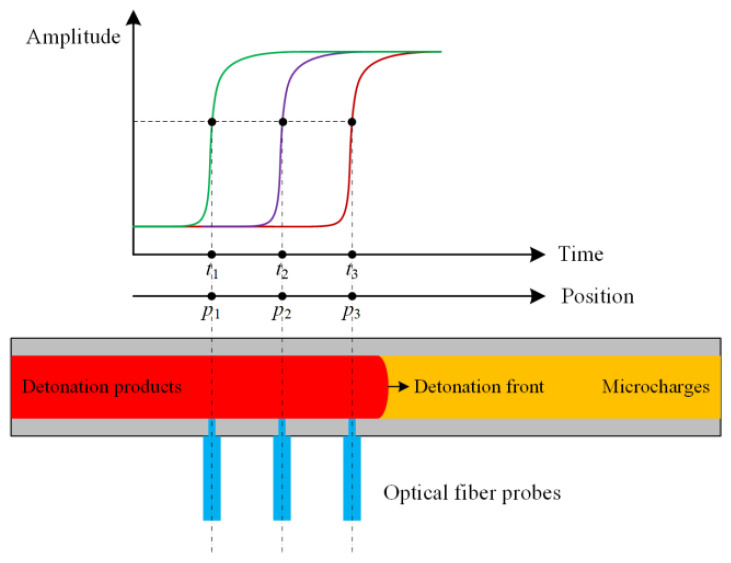
Schematic diagram of data acquisition.

As shown in Figure 9, the data obtained through the above experimental procedures are defined as {(*p_i_*, *t_i_*)}*_i_*_= 1, 2,_…_, *n*_, where *p_i_* and *t_i_* are the probe position and the time when the detonation wave reaches this position, respectively. The detonation velocity of one experiment can be expressed as follows by employing the unitary linearity regression method.

(3) $$D=\frac{\sum_{i=1}^{n}\left(t_{i}-\bar{t}\right)\left(p_{i}-\bar{p}\right)}{\sum_{i=1}^{n}{\left(t_{i}-\bar{t}\right)}^{2}},$$

where $\bar{p}=\frac{1}{n}\sum_{i=1}^{n}p_{i}$ and $\bar{t}=\frac{1}{n}\sum_{i=1}^{n}t_{i}$ are average values of *p_i_* and *t_i_*, respectively. The correlation coefficient reflects the closeness of the linear relationship between variables *p_i_* and *t_i_*, and it takes the form

(4) $$\gamma =\frac{\sum_{i=1}^{n}\left(t_{i}-\bar{t}\right)\left(p_{i}-\bar{p}\right)}{\sqrt{\sum_{i=1}^{n}{\left(t_{i}-\bar{t}\right)}^{2}}\sqrt{\sum_{i=1}^{n}{\left(p_{i}-\bar{p}\right)}^{2}}}.$$

A *t*-distribution significance test must be performed on the correlation coefficient for every experiment to judge whether the calculated detonation velocity is meaningful. The look-up table method is used to simplify the calculation process [14]: (1) determine the degree of freedom *m* and significance level *α*. In this paper, *m* is equal to the number of fiber probes (called *n*) minus 2, and *α* is 0.01. (2) Find the critical value of correlation coefficient named *γ_c_* in Table 4 according to *m* and *α*. In this paper, *n* is set to 3, so *γ_c_* equals 1.000. (3) Compare the absolute value of correlation coefficient *γ* calculated by Equation (4) with *γ_c_*. If *γ* is 1.000 in the case of four significant digits, we think that *t_i_* and *p_i_* have a positive correlation at the significance level of 0.01. Under this condition, the calculated detonation velocity *D* is meaningful. 

If detonation velocity experiment is repeated *m* times, the average detonation velocity can be written in the form

(5) $$\bar{D}=\frac{1}{m}\sum_{j=1}^{m}D_{j}.$$

and its standard deviation has the form

(6) $$\sigma_{D}=\sqrt{\frac{\sum_{j=1}^{m}{\left(D_{j}-\bar{D}\right)}^{2}}{m-1}}.$$

## 4. Results and Discussion

### 4.1. Experimental Results of Different Probe Spacings

**Figure 10:**
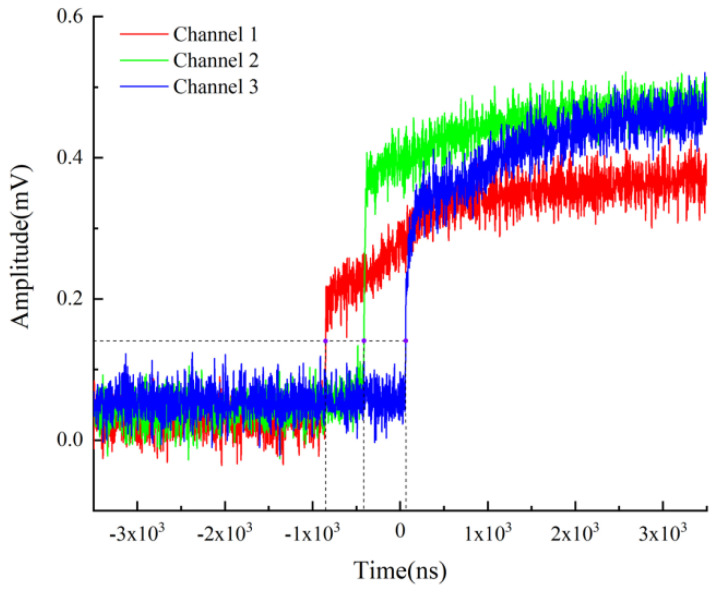
Typical output signal of the oscilloscope.

Usually, the microcharge length is a few centimeters due to the limitation of manufacturing technology. In order to realize the multi-point measurement of detonation velocity in a finite length, it is necessary to study the optimal distance between fiber probes. For discussing the influence of probe spacing on detonation velocity, four groups of experiments were carried out and each group of experiments was repeated three times. In the four groups of experiments, fiber probe spacings were set to 1, 2, 3 and 4 mm, respectively. The typical signals collected by the oscilloscope are shown in Figure 10, which corresponds to the first experiment with conditions of *L* = 4 mm and *a* = 0.5 mm.

The time when the detonation wave reaches the corresponding position was all read at the output voltage of 0.15 mV for reducing the influence of noise signals on reading. The experimental data and the corresponding detonation velocity *D* and correlation coefficient *γ* calculated by Equations (3) and (4) are shown in Table 5, Table 6, Table 7 and Table 8. It can be observed that every calculated detonation velocity *D* is meaningful because its correlation coefficient is 1.000 in the case of four significant digits.

The average detonation velocity $\bar{D}$ and corresponding standard deviation *σ*_D_ of each group of experiments are given in Table 9 by Equations (5) and (6).

### 4.2. Results of Comparative Experiment

In order to verify the feasibility and accuracy of the method applied in this paper, the comparative experiments between ionization probes and fiber probes were performed. In our previous research [15], a flexible ionization-conducted probe sensor was used for measuring detonation velocity produced by a booster explosive which is the same as that employed in this paper. In addition, the initiation mode, charge size, and constraint condition of the explosive are also consistent with this contribution. The experiments were performed under the condition that the spacing between two adjacent ionization probe groups (called *d*) is 1 mm, 2 mm, 4 mm, and 8 mm, respectively. The average detonation velocity and corresponding standard deviation of each group of experiments are given in Table 10.

### 4.3. Discussions

Table 9 shows that the detonation velocity of each group of experiments is basically consistent. However, the standard deviation of *L* = 4 mm is significantly smaller than the others, which may be attributed to the NA of the optical fiber.

As we know, the NA represents the angle range of incident light received by the optical fiber, that is to say, the light in a certain angle range will pass through the fiber and be converted into an electrical signal by the photodetector. Considering the complexity of the detonation process, when the detonation wave propagates to a certain position where is in the light receiving range of the fiber, the oscilloscope may be triggered and generate a step signal. As shown in Figure 3, the uncertainty range of optical signals can be defined as *h*. Under other invariable conditions, the smaller the value of *h*/*L*, the lower the standard deviation of detonation velocity. In this study, the *h* value of each experiment is the same and the *L* value of the fourth group of experiments is the largest. Therefore, the minimum standard deviation of *L* = 4 mm is understandable. Furthermore, based on these experimental results, the value of *h*/*L* should preferably be less than 0.1.

The theoretical detonation velocity can be calculated by employing Explo5 thermo-chemical prediction software and it is approximately 8743 m/s [15]. According to our previous research, *d* = 4 mm is an appropriate choice for the densely arranged ionization probe array. Under this condition, the average detonation velocity of the booster explosive is 8656 m/s and the standard deviation is 57 m/s. Our measurement results are all close to the theoretical value. However, compared with the case of *L* = 4 mm in this paper, the detonation velocity and standard deviation acquired by the ionization probe are small and large, respectively. This occurrence may be due to the difference in measurement mechanism between the two methods.

The ionization probe group at the off-state can respond to an incoming detonation wave with the conduction induced by ionization characteristics of the detonation reaction zone. The reaction zone width can be considered as the uncertainty range of electrical signals and it is less than the value of *h* in this paper [16,17]. The time interval between two probes may become smaller or larger than the ideal conditions because of the existence of uncertainty range. Therefore, the detonation velocity measured by fiber probes may be higher than that by electric probes. However, due to the influence of electromagnetic interference, the standard deviation of detonation velocity measured by ionization probes will be larger than that by fiber probes.

In this study, the total error mainly includes the fiber probe spacing error caused by the base plate fabrication, fiber probe installation, the timing error of the oscilloscope and photodetector, and the reading error. The error of the fiber probe spacing caused by the base plate fabrication can be declined by employing femtosecond laser processing technology. Moreover, the reading error can be improved by employing a time-interval measuring instrument with high sampling clock frequency and measuring the principle of the time to voltage converter method.

## 5. Conclusions and Perspectives

In this paper, we have presented a determination method of detonation velocity of microcharges by employing discrete fiber probes. This method is simple to implement, scalable for multi-channel, and has minimal perturbation to the detonation wave. The measurement system was established, and many detonation velocity experiments were carried out. The experimental results indicate that *L* = 4 mm is a relatively appropriate spacing for densely arranged fiber probes. The comparative experiments show that the fiber probe method is a better choice to measure the detonation velocity of microcharges without considering the cost.

The detonation velocity measurement technology for microcharges is still a pressing subject, this study is a preliminary and effective attempt, and more efforts need to be paid in this research. The method reported here can be used to measure the diameter effect curve. The critical charge dimension of the explosive is obtained by the conical charge method which employs fiber probes with a spacing of 4 mm to measure the propagation trajectory of the detonation wave. Then, under the same experimental conditions except for charge dimension, a series of detonation velocity experiments are performed by the method proposed in this paper. In these experiments, the charge size starts from the critical value and increases gradually in a certain increment. Finally, the diameter effect curve under certain conditions can be acquired by plotting a graph of charge dimension against detonation velocity. This work will be the focus of our follow-up research. Moreover, integration of detonation velocity measuring equipment based on discrete ionization probes and fiber probes is also an interesting research direction. Our further work will be performed in the above aspects to promote the research of microcharges.

## Figures and Tables

**Table 1:** Structural parameters of the optical fiber.

Structure Parameters	Value
Working wavelength/nm	800–1600
NA	0.22
Core diameter/μm	105
Cladding diameter/μm	125
Coating diameter/μm	245
Core-cladding concentricity error/μm	≤3
Working temperature/°C	−55 to 85
Length/m	10

**Table 2:** Performance parameters of the photodetectors.

Model Number	KG-PR-500M-B	FPD310-FC-VIS	DET025AFC/M
Detector type	Si	Si	Si
Spectral range/nm	320–1000	400–1000	400–1100
3 dB bandwidth/MHz	500	5–1000	2000
Rise time/ns	0.6	0.5	0.15
Max. gain	2.5 × 10^3^	2 × 10^4^	—
NEP/pw/√Hz	36	25.5	9.29 × 10^−3^
Optical input	FC	FC/PC	FC/PC
Output coupling	DC	AC	DC
Output connector	SMA	SMA	SMA

**Table 3:** Charge width data of 0.5 mm.

Measured Point	Value/mm
1	0.504
2	0.496
3	0.500
4	0.512
5	0.496
Average value	0.5016

**Table 4:** The relationship between *γ_c_* and *m* when *α* = 0.01.

*m*	*γ_c_*
1	1.000
2	0.990
3	0.959
4	0.917

**Table 5:** Experimental data with *L* = 1 mm, *a* = 0.5 mm.

Serial Number	*t*_1_/ns	*t*_2_/ns	*t*_3_/ns	*D*/m·s^−1^	*γ*
1	38	152	263	8888	1.0000
2	−1	115	223	8925	0.9998
3	−5	108	218	8968	1.0000

**Table 6:** Experimental data with *L* = 2 mm, *a* = 0.5 mm.

Serial Number	*t*_1_/ns	*t*_2_/ns	*t*_3_/ns	*D*/m·s^−1^	*γ*
1	81	313	532	8867	0.9999
2	−120	114	335	8789	0.9999
3	17	237	466	8907	0.9999

**Table 7:** Experimental data with *L* = 3 mm, *a* = 0.5 mm.

Serial Number	*t*_1_/ns	*t*_2_/ns	*t*_3_/ns	*D*/m·s^−1^	*γ*
1	−28	296	652	8817	0.9996
2	7	356	690	8783	0.9999
3	−455	−119	224	8836	1.0000

**Table 8:** Experimental data with *L* = 4 mm, *a* = 0.5 mm.

Serial Number	*t*_1_/ns	*t*_2_/ns	*t*_3_/ns	*D*/m·s^−1^	*γ*
1	−851	−414	58	8796	0.9998
2	−349	107	558	8820	1.0000
3	−1607	−1163	−698	8799	0.9999

**Table 9:** Average value and standard deviation of detonation velocity experiment in this paper.

*L*/mm	$\bar{D}$/m·s^−1^	*σ_D_*/m·s^−1^
1	8927	40
2	8854	60
3	8812	27
4	8805	13

**Table 10:** Average value and standard deviation of previous detonation velocity experiment.

*d*/mm	$\bar{D}$/m·s^−1^	*σ_D_*/m·s^−1^
1	8618	213
2	8886	121
4	8656	57
8	8693	52

## Data Availability

Data are contained within the article.
